# Effect of Lokomat^®^ Robotic Rehabilitation on Balance, Postural Control, and Functional Independence in Subacute and Chronic Stroke Patients: A Quasi-Experimental Study

**DOI:** 10.3390/medsci13030157

**Published:** 2025-08-28

**Authors:** Marina Esther Cabrera-Brito, María del Carmen Carcelén-Fraile, Agustín Aibar-Almazán, Fidel Hita-Contreras, Paulino Vico-Rodríguez, Marta Cano-Orihuela, Yolanda Castellote-Caballero

**Affiliations:** 1 Department of Health Sciences, Faculty of Health Sciences, University of Jaén, 23007 Jaén, Spain; 2 Island Rehabilitation Institute, S.L. Las Palmas de Gran Canaria, Spain; 3 Department of Educational Sciences, Faculty of Social Sciences, University of Atlántico Medio, 35017 Las Palmas de Gran Canaria, Spain; 4 Department of Health Sciences, Faculty of Health Sciences, University of Atlántico Medio, 35017 Las Palmas de Gran Canaria, Spainyolanda.castellote@atlanticomedio.es (Y.C.-C.)

**Keywords:** robotic rehabilitation, balance, postural control, functional independence, neurorehabilitation, neurological disorders

## Abstract

**Background/Objectives**: Balance, postural control, and functional independence are essential components for the autonomy of people with neurological conditions. Robotic technologies such as the Lokomat^®^ have emerged as promising tools in rehabilitation, but their effectiveness when integrated into functional programs requires further evidence. The objective of this study was to evaluate the impact of an intensive robotic intervention on these three functional variables. **Methods**: A single-group, quasi-experimental pretest–posttest study was conducted with 136 participants who received a robotic rehabilitation intervention using the Lokomat^®^ device, and focused on functional tasks over several weeks. Balance (using the Berg scale), postural control (using the PASS), and functional independence (using the Barthel index) were assessed, comparing pre- and post-intervention results using parametric and non-parametric tests. **Results**: The results showed statistically significant improvements in all three variables after the intervention. The mean Berg score increased from 11.76 to 21.91 points (*p* < 0.001), postural control increased from 15.53 to 21.90 points (*p* < 0.001), and the Barthel index increased from 24.71 to 41.76 points (*p* < 0.001). In all cases, the effect sizes were large (d > 0.90). **Conclusions**: A rehabilitation program including intensive, task-oriented Lokomat^®^ training was associated with improvements in balance, postural control, and functional independence. Given the single-group design without a control arm, these findings reflect associations and do not establish causality.

## 1. Introduction

Stroke, also known as cerebrovascular accident (CVA), is a clinical syndrome characterized by the sudden interruption of cerebral blood flow, either due to an occlusion (ischemic stroke) or hemorrhage (hemorrhagic stroke), causing focal or global neuronal damage, depending on the magnitude and location of the event [1]. According to the World Health Organization (WHO), stroke is the second leading cause of death and the leading cause of acquired disability in adults worldwide [2]. Most patients who survive a stroke experience neurological sequelae of varying severity, with motor, sensory, cognitive, and balance impairments being particularly prevalent [3]. These sequelae can significantly limit the individual’s ability to perform activities of daily living (ADL), affecting their autonomy and functional independence [4].

Among the most disabling motor consequences is loss of balance, both in static and dynamic conditions [5]. Balance is defined as the body’s ability to maintain the center of mass (CM) within the base of support (BOS) in order to maintain a stable posture, both at rest and in motion [6]. This complex process requires the integration and coordination of the visual, vestibular, somatosensory, and musculoskeletal systems, as well as adequate central processing in structures such as the brainstem, cerebellum, and motor cortex [7]. After a stroke, balance is compromised due to muscle weakness, altered postural tone, loss of proprioception, and dysfunction in automatic postural reflexes [8]. As a result, the patient becomes more susceptible to falls, their ability to move safely is diminished, and their overall functional performance is impaired [9].

Closely related to balance is postural control, which refers to the nervous system’s ability to regulate the body’s position in space to maintain stability and body orientation [10]. This postural control involves both anticipatory and reactive mechanisms that allow the muscles to adjust in response to external or internal disturbances [11]. After a stroke, alterations are observed in the synergistic activation of muscle groups, trunk alignment, movement strategy (e.g., over support on the healthy side of the body), and neuromuscular recruitment during motor tasks [12]. Impaired postural control limits the development of efficient and functional gait patterns, preventing the patient from achieving independent and safe locomotion [13].

This loss of postural control and balance has a direct impact on the patient’s functional independence [14]. Functional independence is understood as the ability to independently perform everyday tasks such as ambulation, toileting, dressing, eating, and mobility in the environment [15]. Several studies have shown that the quality of balance is a strong predictor of the level of functional independence in people with stroke. In fact, scales such as the Berg Balance Scale (BBS), the Timed Up and Go Test (TUG), and the Functional Independence Measure (FIM) are commonly used to assess clinical progress and establish functional prognoses. Therefore, the recovery of balance and postural control becomes a priority within the rehabilitation process [16,17].

Conventional neurological physiotherapy constitutes the fundamental pillar of the therapeutic approach for patients with post-stroke sequelae [18]. This intervention is based on principles of neuroplasticity, motor learning, and motor control, and seeks to promote functional recovery through techniques such as balance training, muscle strengthening exercises, postural reeducation, sensorimotor stimulation, and specific tasks oriented toward ADLs [19]. Classical approaches include methods such as Bobath, Perfetti, PNF, and treadmill or parallel bar training [20]. Recent research, such as the cross-sectional study conducted in Catalonia, has mapped physiotherapy approaches for stroke survivors, providing an updated overview of clinical practice patterns and highlighting the need for integrated and standardized rehabilitation strategies [21]. However, although conventional physical therapy has proven effective in improving functional capacities, it has certain limitations when it comes to stimulating large volumes of intensive, repetitive, and highly specific exercise, which are essential elements for inducing significant changes in the damaged nervous system [22].

It is in this context that the implementation of new technology-based therapeutic strategies becomes relevant, complementing and enhancing conventional interventions [23]. Robotic rehabilitation has emerged in the last two decades as an innovative tool for the neurorehabilitation of stroke patients. It is based on the use of electromechanical or robotic devices that assist and guide the patient’s movement in a precise, repetitive, and programmable manner, allowing for a high volume of specific motor practice [24]. Furthermore, many of these devices incorporate visual, auditory, or haptic feedback, which increases patient motivation and engagement during therapy. Robotic rehabilitation has shown positive results in improving gait, balance, postural control, and overall functionality, especially in the subacute and chronic phases of stroke [25].

One of the most representative devices within robotic rehabilitation is the Lokomat^®^, developed by Hocoma [26]. This system consists of a robotic lower limb exoskeleton coupled to a treadmill and a body-weight suspension system, enabling gait training under highly controlled conditions [27]. The Lokomat^®^ is equipped with motors that guide the hip and knee joints, allowing it to reproduce physiological gait patterns. The body weight load can be progressively adjusted, and movement parameters, such as speed, amplitude, and assistance level, are fully customizable. It also includes real-time biofeedback systems, allowing the patient to interact with virtual environments or therapeutic games during gait, promoting active motor learning [28]. Beyond commercial exoskeletons, recent developments in lower-limb rehabilitation robotics, including parallel-robot architectures with dedicated control systems, have undergone functional validation for stroke rehabilitation, underscoring the rapid evolution of robot-assisted platforms [29]. In addition, the integration of augmented reality and serious gaming into robot-assisted gait therapy has been proposed to enhance engagement and motor learning through interactive, goal-oriented tasks and real-time feedback, aligning with the biofeedback/gamified training used in our program [30].

Several studies have shown that the use of the Lokomat^®^ can improve parameters such as gait symmetry, muscle activation, energy efficiency of movement, and patient confidence in their functional capacity [31]. Furthermore, it has been observed that, by providing a safe and repetitive environment, the Lokomat^®^ can facilitate cortical and spinal reorganization, contributing to motor recovery after neurological damage [32]. In post-stroke patients, its application has been particularly useful in addressing alterations in dynamic balance and postural control during locomotion, key elements for achieving satisfactory functional independence [33].

In addition, recent systematic reviews and meta-analyses have consolidated the role of the Lokomat^®^ in stroke rehabilitation, reporting significant improvements in lower limb motor function, walking speed, and independence in activities of daily living compared to conventional therapy alone. These studies also emphasize the device’s potential to enhance neuroplasticity and patient engagement through intensive, repetitive, and task-specific training [34,35]. Evidence suggests that optimal results are obtained when the Lokomat^®^ is integrated into multidisciplinary rehabilitation programs and used in conjunction with conventional physiotherapy rather than as a stand-alone intervention. Moreover, clinical guidelines for neurorehabilitation are increasingly recognizing the importance of robotic-assisted gait training, including Lokomat^®^, as an evidence-based option for selected stroke patients [36].

Therefore, the objective of this study is to evaluate the effect of a robotic rehabilitation program using the Lokomat^®^ device on balance, postural control, and functional independence in stroke patients.

## 2. Materials and Methods

### 2.1. Study Design and Participants

This study was conducted using a quasi-experimental pretest–posttest design with a single group, with no control group, at the Hospital ICOT Ciudad de Telde, Gran Canaria, where all participants were recruited. This type of design allows for the analysis of the effects of a specific intervention, in this case, robotic rehabilitation using the Lokomat^®^ (Hocoma, Volketswil, Switzerland) device, on clinical variables of interest, such as balance, postural control, and functional independence, comparing the values obtained before and after the intervention. The study was approved by the Ethics Committee of the University of Atlántico Medio (protocol code CEI/05-018) and was registered at ClinicalTrials.gov (identifier NCT06840366) prior to its commencement. Prior to the start of the intervention, all participants (or their legal representatives, if necessary) were asked to sign an informed consent form. All procedures were carried out following ethical and confidentiality principles, in accordance with the Declaration of Helsinki (Fortaleza, Brazil, 2024).

For selection, participants had to meet the following inclusion criteria: (i) Confirmed diagnosis of stroke. (ii) Age ≥ 18 years (no upper age limit). (iii) Sufficient cognitive level to follow simple instructions (Mini-Mental State Examination ≥ 24). (iv) Medical and hemodynamic stability to participate in intensive physical therapy sessions. (v) Signature of informed consent by the patient or legal guardian. However, participants were excluded if they met any of the following exclusion criteria: (i) Diagnosis of an additional neurological condition (e.g., Parkinson’s disease, multiple sclerosis, spinal cord injury). (ii) Metallic implants or electronic devices incompatible with the use of the Lokomat^®^. (iii) Cardiovascular instability or medical contraindications to moderate physical activity. (iv) Simultaneous participation in other robotic rehabilitation programs or clinical studies.

### 2.2. Recruitment and Sampling

We used consecutive sampling of all adults with a confirmed stroke diagnosis who were referred to and admitted/attended the rehabilitation service at Hospital ICOT Ciudad de Telde, Gran Canaria, during the study period. Potential candidates were identified from daily admission lists, outpatient schedules, and referrals from the referring hospitals’ neurology/rehabilitation services. Screening against the predefined eligibility criteria was performed by trained clinicians using the medical record and a standardized checklist. Eligible patients (or legal representatives) were approached in person, received written study information, and provided written informed consent prior to baseline assessment. Reasons for exclusion or refusal (e.g., additional neurological conditions, contraindications to Lokomat^®^, cardiovascular instability, concurrent robotic programs, or cognitive ineligibility) were documented in a screening log. To reduce selection bias, no socioeconomic or sex restrictions were applied, and language requirements were limited to ensuring adequate comprehension per the cognitive criterion (MMSE ≥ 24). No financial incentives were offered. The sociodemographic and clinical profile of the enrolled sample is summarized in Table 1 to support appraisal of representativeness.

### 2.3. Outcomes

To evaluate the effects of robotic intervention in stroke patients, three standardized and clinically validated instruments were used, all highly sensitive to change in neurological rehabilitation settings. Measurements were taken at two time points: before starting the intervention (pretest) and after completing the robotic rehabilitation program (posttest), to assess functional changes attributable to the treatment. To limit detection bias, outcome assessments were performed by trained clinicians following standardized written instructions and scoring manuals for each instrument. Assessors used the same instrument versions at pre- and posttest, and were instructed to adhere to fixed testing scripts.

#### 2.3.1. Balance

This outcome represents functional balance during standardized tasks. The Berg Scale is a widely used tool for assessing static and dynamic balance in adults with neuromotor impairments [37]. It consists of 14 items that assess various functional tasks, such as standing, transferring, trunk rotation, and turning gait. Each item is scored on an ordinal scale from 0 to 4, with 0 indicating the lowest functional capacity and 4 indicating independent and safe performance of the task, with a maximum total score of 56 points. Scores below 45 are associated with an increased risk of falls. This scale has demonstrated high interobserver reliability (ICC > 0.95) and concurrent validity in stroke patients.

#### 2.3.2. Postural Control

This outcome represents trunk alignment and control of body position (anticipatory/reactive) across positions. The Postural Assessment Scale for Stroke Patients (PASS) is specifically designed to assess postural control in stroke patients, both in the acute and chronic phases [38]. It includes 12 items that assess postural maintenance and changes in different positions (recumbent, sitting, and standing). Each item is scored from 0 to 3, with a maximum total score of 36 points. The PASS is particularly sensitive for detecting small functional changes during the early stages of post-stroke recovery and has demonstrated good discriminative and predictive validity for the evolution of postural control and gait. Its use is recommended for monitoring clinical progress in the rehabilitation setting.

#### 2.3.3. Functional Independence

The Barthel Index is a commonly used instrument for measuring functional independence in basic activities of daily living (BADL) [39]. It assesses a patient’s ability to perform 10 activities, including feeding, dressing, toileting, mobility, and climbing and descending stairs, among others. Scoring varies by task, with a maximum of 100 points awarded, with higher scores indicating greater autonomy. The BI is sensitive to clinical changes after rehabilitation interventions and has high reliability (α > 0.90). It is widely used in clinical research with neurological populations, including post-stroke patients.

### 2.4. Sample Size Calculation

The sample size was calculated using G*Power software (version 3.1.9.7) for a quasi-experimental design with repeated measures (pretest–posttest). A small effect size (d = 0.25), a statistical significance level of α = 0.05, and 80% power (1 − β = 0.80) were assumed, considering a two-sided distribution. Under these parameters, the necessary sample size was estimated to be 128 subjects. Anticipating a 5% attrition rate (≈7 participants), we set a target sample of *n* ≈ 135. Ultimately, 136 participants were enrolled and completed the study (0% attrition), exceeding the target and preserving the planned power.”

### 2.5. Intervention

The intervention consisted of a robotic rehabilitation program using the Lokomat^®^ system (Hocoma AG, Switzerland), aimed at improving gait, dynamic balance, postural control, and overall functionality in patients with neurological impairment. This device combines an automated robotic lower extremity orthosis with a treadmill and a partial body weight support (BWS) system, allowing for safe and repeatable reproduction of the physiological gait pattern. Lokomat^®^ sessions were delivered as part of an integrated rehabilitation plan; participants also attended conventional physiotherapy and/or occupational therapy as clinically indicated.

Each session began with patient preparation, including individualized placement and adjustment of the suspension harness, followed by attachment of the robotic orthosis to the hips, knees, and ankles. Special attention was paid to biomechanical alignment in the frontal and sagittal planes, adjusting segment lengths, pivot points, and joint centering to minimize postural compensations and ensure proper gait kinematics. The key training parameters were then calibrated: (i) Treadmill speed refers to the belt speed (i.e., the patient’s walking speed while in the device). During treatment it was adjusted within 0.8–2.5 km/h, increasing or decreasing in 0.1–0.3 km/h steps according to (a) maintenance of a stable gait pattern (no knee collapse in stance or toe drag in swing); (b) therapist-rated gait quality (symmetry, step length, cadence); and (c) physiological tolerance (see criteria below). If loss of pattern, pain, or marked spastic co-contraction appeared, speed was reduced and/or a brief rest was given. (ii) Tolerance was monitored every 5–10 min using perceived exertion (Borg CR10 target 3–5), heart rate within a clinician-defined safe zone (≈≤60 to 70% of age-predicted maximum), and observation of fatigue/compensations. Progress was defined as meeting all of the following in two consecutive sessions at a given setting: correct device-guided gait without manual aid, absence of knee buckling or toe drag, and Borg CR10 ≤ 5 with stable vitals. When these conditions were met, speed was increased by 0.1–0.3 km/h and/or BWS was reduced. Session intensity was eased if Borg CR10 ≥ 7, pain ≥ 5/10, SpO_2_ < 92%, or unsafe pattern. (iii) BWS is the proportion of body weight unloaded by the harness (displayed by the device). The initial prescription was 40–60%, individualized to ensure safe knee extension in stance and adequate foot clearance. BWS was reduced by 5–10% when the patient maintained for ≥2 min: (a) knee stability in stance without manual aid; (b) step-length asymmetry within ~10%; and (c) no increase in compensatory trunk movements. The target by program end was ≤20% whenever feasible; otherwise, the highest functionally safe level was maintained. (iv) Level of robotic assistance (from full control to an actively assisted mode). Robotic assistance and pattern control. Guidance at hip and knee started higher and was progressively shifted toward actively assisted modes as voluntary effort increased. (v) ROM and guidance force were tuned to minimize compensations while allowing patient-initiated movement.

The training was structured in three sequential phases, adapted to the patient’s functional level: (i) Safe gait phase: In this first stage, the Lokomat^®^ fully guides the patient’s movement, without requiring voluntary muscle activation. It is primarily used for initial neuromuscular adaptation, motor pattern learning, and familiarization with the robotic environment. (ii) Progressive physiological gait phase: The level of robotic assistance and weight-bearing are gradually decreased, with the goal of promoting greater active patient participation, increasing voluntary motor control, and strengthening functional muscle activation. Specific tasks that challenge gait stability and rhythm are incorporated. (iii) Functional task-oriented gait phase (visual biofeedback, therapeutic games, dual tasks). In the final phase of each session, patients trained their gait with real-time visual biofeedback and therapeutic games targeting step-length symmetry, rhythmicity, and weight shift. Examples include target-stepping, symmetric step-length tracing, cadence/rhythm matching, and obstacle-avoidance tracks. Cognitive dual tasks (e.g., counting backwards by 3 s, alternating letter–number sequences, category naming) and motor dual tasks (e.g., reacting to visual cues while stepping) were added to challenge attentional control and executive function during walking. Progression was achieved by increasing belt speed, reducing BWS, raising game difficulty (smaller targets/faster cues), or increasing dual-task complexity. When available, the FreeD^®^ pelvis module was activated to allow controlled lateral/transverse pelvic motion and enhance dynamic postural control during weight shift. Additionally, the FreeD^®^ module was used, an extension of the Lokomat^®^ that allows active mobilization of the pelvis in the lateral and transverse planes, simulating the physiological shift of the center of mass. This function promotes training in dynamic postural control, trunk activation, and balance stabilization during the gait cycle. Each session lasted 1 h, with a frequency of 2 sessions per week for a period of 10 weeks. Coordination with conventional therapy. To enhance transfer, Lokomat^®^ gait training was scheduled in coordination with therapist-led conventional sessions targeting balance, transfers, and task-oriented ADL practice. The clinical team aligned session goals (e.g., weight shift, step-length symmetry, cadence) so that conventional therapy could capitalize on the gait pattern, cueing, and real-time biofeedback practiced in the device. Within the 60 min session, setup/warm-up took ~5 min, 40–45 min was devoted to gait training (including the task-oriented phase), and 5–10 min to cool-down. Short standing rests (30–120 s) were allowed as needed to preserve pattern quality and safety. To mitigate performance bias, the same treatment protocol and progression rules were applied to all participants, and therapists followed standardized operating procedures for device setup, parameter tuning, and safety checks. Lokomat^®^ sessions were coordinated with the patients’ conventional physiotherapy to maximize carry-over. Typically, gait training in the device was scheduled immediately before therapist-guided units addressing balance, transfers, and task practice (ADLs). The same clinical team aligned session goals (e.g., weight shift, step-length symmetry, cadence) and used standard operating procedures for device setup, parameter progression, and safety, so that conventional therapy could directly capitalize on the gait pattern, cueing, and biofeedback practiced in the robot. Continuous monitoring was performed during each session to monitor fatigue, possible discomfort, signs of overexertion, and maintain safety parameters. In addition to the Lokomat^®^ sessions, participants continued to receive routine multidisciplinary rehabilitation at the study facility (e.g., conventional physiotherapy and/or occupational therapy) as clinically indicated. These concomitant therapies were not standardized or controlled within the study protocol.

The protocol was applied individually and progressively, dynamically adjusting parameters based on the patient’s functional progress. This approach allowed us to work under the principles of intensity, repetition, specificity, and motor variability, which are essential for inducing neuroplasticity, improving motor learning, and facilitating the functional transfer of gait to the real-life environment.

### 2.6. Statistical Analysis

In the present study, which featured a pretest–posttest design with a single group and no control group, the statistical analysis aimed to determine whether there was a significant difference between the pre- and post-intervention measurements. To do so, the process began with a descriptive analysis of the data, including the calculation of measures of central tendency and dispersion (mean and standard deviation) for the variables at the two measurement points. The Kolmogorov–Smirnov test was then used to verify compliance with the assumption of normality. If the data were normally distributed, a related samples test was applied. Otherwise, nonparametric tests such as the Wilcoxon signed-rank test were used. In all analyses, a *p*-value < 0.05 (two-tailed) was considered statistically significant. All analyses were performed using IBM SPSS Statistics (version 20). The statistical approach was pre-specified: two-tailed α = 0.05, reporting 95% confidence intervals and effect sizes for change scores. Given potential non-normality, we complemented the paired t-test with the Wilcoxon signed-rank test as a confirmatory analysis.

## 3. Results

A total of 136 participants completed the study. The sample consisted of patients diagnosed with stroke who met the inclusion criteria and completed the full robotic rehabilitation program using the Lokomat^®^ device. The mean age of the participants was 61.57 ± 1.11 years, with an age range between 23 and 93 years. Of the total, 65 (47.80%) were women and 71 (52.20%) were men. Table 1 presents the general sociodemographic and clinical characteristics of the sample, including distribution by sex, age, lesion laterality, and time since the stroke. This information allows for an adequate contextualization of the study population and its representativeness with respect to routine clinical practice in neurological rehabilitation (Table 1).

### 3.1. Balance

The balance variable, assessed using the Berg scale, showed a notable increase in scores after the intervention. At the pre-intervention level, the mean was 11.76 points (standard deviation = 13.99), with a 95% confidence interval between 9.38 and 14.13. The median was 6.00, and values ranged from 0 to 55, with an interquartile range of 14 points, indicating high dispersion. The distribution was positively skewed (skewness = 1.507), with a kurtosis of 1.246, suggesting a concentration of low scores with high extreme values. At the post-intervention level, the mean increased to 21.91 points (standard deviation = 18.09), with a 95% confidence interval between 18.84 and 24.98. The median also increased, reaching 16.50 points, and the range widened slightly to a maximum of 56. The post-intervention distribution was more symmetrical (skewness = 0.574) and with lower kurtosis (–1.073), indicating an improvement in dispersion and a greater concentration of values around the mean. These results suggest a significant improvement in postural control after the intervention. The normality of the variable was assessed using the Kolmogorov–Smirnov test, and statistically significant results were obtained (*p* < 0.001), indicating that the data do not follow a normal distribution.

A paired-samples t-test was performed to analyze whether there were significant differences between the pre- and post-intervention values on the Berg scale. The results showed a mean difference of −10.15 points (SD = 10.79; standard error = 0.93), indicating an improvement in the post-intervention values. The 95% confidence interval for the difference ranged from −11.98 to −8.33, reinforcing the consistency of the observed change. The test was statistically significant (t (135) = −10.980, *p* < 0.001), so it is concluded that there was a significant improvement in the functional balance of the participants after the intervention, as measured by the BERG scale (Table 2).

Due to the lack of normality in the variable data, the nonparametric Wilcoxon signed-rank test for related samples was applied to compare the scores obtained before and after the intervention. The results indicated a statistically significant difference between the two measurements (*p* < 0.001), which led to the rejection of the null hypothesis of equal medians. Therefore, it is concluded that the intervention produced a significant change in the levels of functional balance measured by the BERG scale.

### 3.2. Postural Control

On the assessment of postural control using the PASS scale, participants obtained a mean score of 15.53 points (SD = 9.23) at the pre-intervention level, with a 95% confidence interval between 13.96 and 17.10. The median was 15.50, and scores ranged from 0 (minimum) to 35 (maximum), with an interquartile range of 14. The distribution was approximately symmetrical (skewness = 0.115) and without excess kurtosis (kurtosis = −0.820), suggesting a moderately normal distribution for this measure. After the intervention, PASS scale scores increased to a mean of 21.90 points (SD = 9.37), with a 95% confidence interval between 20.31 and 23.49. The median increased to 23.50, indicating generalized improvement. The range was maintained between 1 and 36, with an interquartile range of 15. The post-intervention distribution also remained free of significant asymmetries (skewness = −0.350; kurtosis = −0.950).

The normality assumptions for the scores obtained on the PASS scale were analyzed. The result was not statistically significant (D = 0.072; *p* = 0.081), indicating that the null hypothesis of normality was not rejected. Therefore, it can be assumed that the postural control data from the pre-test follow an approximately normal distribution.

A paired-samples *t*-test was applied to compare the pre- and post-intervention scores on the PASS scale, used to assess participants’ postural control. The results indicated a mean difference of −6.37 points (standard deviation = 4.88; standard error of the mean = 0.42), with a 95% confidence interval between −7.20 and −5.54. The test was statistically significant (t (135) = −15.206, *p* < 0.001), indicating a significant improvement in the balance of the participants after the intervention, according to the values obtained on the PASS scale (Table 2).

### 3.3. Functional Independence

On the assessment of postural control using the Barthel scale, participants obtained a mean score of 24.71 points (SD = 22.82) at the pre-intervention level, with a 95% confidence interval between 20.84 and 28.58. The median was 17.50, and scores ranged from 0 (minimum) to 95 (maximum), with an interquartile range of 95. The distribution showed positive skewness (0.940) and mild kurtosis (0.348). After the intervention, the mean increased to 41.76 points (SD = 28.04; 95% CI 37.01–46.52), with a median of 40.00, a range of 0–100, and IQR = 50. The post-intervention distribution remained approximately symmetric (skewness = 0.322; kurtosis = −0.990).

The normality of the Barthel Index distributions was examined using the Kolmogorov–Smirnov test with Lilliefors correction. Both pre- and post-intervention scores deviated significantly from normality (Barthel_pre: D = 0.165, *p* < 0.001; Barthel_post: D = 0.115, *p* < 0.001), a pattern corroborated by Shapiro–Wilk (pre: W = 0.887, *p* < 0.001; post: W = 0.949, *p* < 0.001). Accordingly, the primary inferential analysis for the Barthel Index was performed with the Wilcoxon signed-rank test (also *p* < 0.001), while the paired-samples t-test is reported for completeness. A paired-samples t-test was applied to compare the pre- and post-intervention scores on the Barthel scale, used to assess participants’ functional independence. The results indicated a mean difference of −17.06 points (standard deviation = 16.39; standard error of the mean = 1.41), with a 95% confidence interval between −19.84 and −14.28. The test was statistically significant (t (135) = −12.136, *p* < 0.001), indicating a significant improvement in the functional independence of the participants after the intervention, according to the values obtained on the Barthel scale (Table 2).

## 4. Discussion

The aim of this study was to analyze the effects of the Lokomat^®^ on balance, postural control, and functional independence in a sample of 136 participants. The results showed statistically significant improvements in all measured variables: balance (assessed with the BERG score), postural control (measured using the PASS), and functional independence (using the Barthel index). Although large and statistically significant gains were observed, the absolute post-intervention means (BBS ≈ 22/56; PASS ≈ 22/36; Barthel ≈ 42/100) indicate persistent balance impairment and severe dependence in basic ADLs. Therefore, the improvements should be interpreted as partial functional gains within a multidisciplinary rehabilitation program rather than restoration of independence or community ambulation during the study window. Our findings align with prior studies reporting benefits of Lokomat^®^-assisted rehabilitation on gait and lower-limb function in stroke (e.g., improvements in gait symmetry and functional outcomes) and with systematic reviews/meta-analyses supporting its clinical utility [26,28]. Beyond gait metrics, the present study adds evidence of balance (BBS), postural control (PASS), and ADL independence (Barthel) gains in a large, heterogeneous clinical cohort, complementing trials that focused primarily on spatiotemporal gait parameters. Importantly, although effect sizes were large, the absolute scores remained compatible with relevant dependency, indicating partial functional gains rather than full independence within the study window. Taken together, these results support Lokomat^®^ as a useful adjunct within multidisciplinary neurorehabilitation while underscoring the need for controlled trials with gait-specific outcomes to confirm effectiveness and define responders. The pattern of results is consistent with a synergistic interaction between high-dose, task-specific robotic gait training and therapist-led conventional sessions targeting balance and ADLs. Real-time biofeedback and rhythmic guidance in the device likely prime trunk–pelvic control and step symmetry, which therapists then consolidate through overground practice, transfers, and functional tasks. The convergence of gains across BBS, PASS, and Barthel supports the coherent, cross-domain impact expected from this integrated approach

Balance is a fundamental motor skill that allows the body to maintain stability, both at rest and in motion. Its deterioration is associated with an increased risk of falls and a loss of autonomy in people with neurological or musculoskeletal disorders [40]. In our study, the results reflected a significant increase in balance scores after the intervention. The mean increased from 11.76 points in the pretest to 21.91 points in the posttest, with a significant difference (t (135) = −10.98, *p* < 0.001) and a large effect size (d = 0.94). This finding indicates a notable improvement in participants’ ability to maintain posture and perform tasks requiring stability. Recent studies show that robot-assisted devices improve balance after stroke. The meta-analysis by Molteni et al. [41] supports gains in stability and function. Likewise, Liao et al. [42] observed significant improvements in balance scores after eight weeks of training with a robotic partial body weight support system. These results support and strengthen the evidence that technological interventions such as the one used in the present study play a key role in the recovery of functional balance. Our results align with Sánchez-Herrera et al. [43], who reported similar gains after sensorimotor training. Studies such as that by Oliveira et al. [44] have also shown that dynamic and static balance programs produce functional improvements in patients with neurological pathologies. Unlike more general interventions, our protocol was characterized by being intensive, functional, and adapted to the deficits observed in the initial assessment. Improvements > 8 points in BBS are clinically significant [45]. We observed >10 points, reinforcing clinical relevance. Our effect size (d = 0.94) exceeds many reports from shorter programs. This likely reflects the protocol’s personalized and progressive design.

Postural control is the body’s ability to maintain trunk alignment and stability in the face of internal or external forces, both in a static position and during movement. It is essential for performing functional activities such as sitting, standing, turning, or transferring from one surface to another [46]. In this study, the results showed a significant improvement in postural control after the intervention, with the mean score increasing from 15.53 points in the pretest to 21.90 in the posttest. The difference was statistically significant (t (135) = −15.21, *p* < 0.001), with a large effect size (d = 1.30). These data reflect a significant improvement in participants’ functional ability to maintain and adjust body posture during daily activities. Similar results were found by Tyson and Connell [47], who showed that progressive postural training significantly contributes to improving trunk stability and reducing the risk of falls. Verheyden et al. [48] also suggest that postural control is directly related to overall functional recovery in patients with neurological injury. Current evidence supports a role for robotics in postural control. Lee et al. [49] showed better trunk control with exoskeleton + physiotherapy, and Calabrò et al. [50] linked robotics with enhanced cortical activation and balance. Our findings confirm effectiveness and exceed minimal clinically relevant thresholds, underscoring clinical impact. The magnitude of change suggests postural adaptation and neuromuscular re-education, yielding meaningful functional gain. Therefore, it can be stated that postural control is a variable highly sensitive to rehabilitation intervention, especially when adaptive robotic technology is integrated, allowing for repetitive, safe, and performance-adjusted training. This reinforces the need to include specific postural control strategies within multidimensional rehabilitation programs.

Functional independence is an essential component of rehabilitation, as it determines the degree of autonomy a person has to perform basic activities of daily living (BADL), such as feeding, grooming, dressing, and getting around [51]. Its improvement not only reflects physical progress but also has a direct impact on the patient’s quality of life, caregiver burden, and social reintegration [52]. In the present study, a statistically significant improvement in functional independence levels was observed after the intervention. The mean Barthel index increased from 24.71 points at pretest to 41.76 at posttest, with a significant difference (t (135) = −12.14, *p* < 0.001) and a large effect size (d = 1.04). This improvement suggests that the intervention had a real impact on participants’ ability to function more independently in their daily routines. This finding indicates that the intervention led to substantial improvements in participants’ overall functionality. Current studies, such as that by Khan et al. [53], have shown that intensive rehabilitation programs incorporating robotics produce superior benefits in functional independence compared to conventional treatments. Likewise, the meta-analysis by Molteni et al. [54] supports that the use of exoskeletons significantly improves patients’ ability to perform daily functional activities, especially when integrated into task-focused programs. Similarly, Lloréns et al. [55] highlighted that virtual reality and enriched environments, when combined with robotic devices, favor the transfer of trained skills to the real environment, with evident improvements in the Barthel index score. Meanwhile, Sale et al. [56] demonstrated that the use of lower limb exoskeletons can shorten hospital stay times by accelerating the recovery of basic functionality. Our >17-point average gain exceeds many reports. The individualized, functional, and intensive protocol likely contributed. This data reinforces the argument that personalized, technology-based approaches represent an effective way to promote autonomy and reduce dependency in patients with neurological impairment.

Despite the positive results obtained, this study has some limitations that should be taken into account when interpreting the findings. First, the quasi-experimental design without a control group limits the possibility of establishing direct causal relationships between the intervention and the observed improvements. Furthermore, the lack of randomization may have introduced selection bias. Another relevant limitation is the lack of medium- and long-term follow-up, which prevents the evaluation of the sustainability of the effects obtained after the intervention. Furthermore, although validated instruments were used to measure the main variables, psychological or motivational aspects that could have influenced treatment response were not explored. Furthermore, baseline stroke severity indices (e.g., NIHSS, mRS) and patient-reported outcome measures (e.g., EQ-5D) were not collected, which limits the characterization of the sample and the patient-centered interpretation of the findings. In addition, the lack of a control arm prevents ruling out placebo/Hawthorne effects, regression to the mean, or residual spontaneous recovery. Outcome assessors were not blinded, which may introduce measurement bias. These factors further limit causal attribution to the Lokomat^®^ component of the program. Finally, gait-specific outcomes (e.g., functional ambulation categories, 10 m walk test, 6 min walk test) were not recorded, limiting direct inferences about ambulation ability despite observed changes in balance and activities of daily living (ADLs). Finally, because the intervention focused exclusively on a controlled clinical setting, the results may not be fully generalizable to other healthcare settings or populations with different characteristics.

### Future Research

Building on these findings, we plan a two-phase research program. Phase 1 will be a randomized controlled trial comparing an integrated rehabilitation model including Lokomat^®^ plus conventional therapy versus conventional therapy alone, with stratification by stroke stage (subacute vs. chronic) and baseline severity. The protocol will (i) standardize and log the dose of all co-interventions; (ii) specify progression rules for treadmill speed, BWS, and guidance; and (iii) use blinded assessors. Primary outcomes will include gait-specific measures (e.g., Functional Ambulation Categories, 10-Meter Walk Test, 6-Minute Walk Test). Secondary outcomes will include balance (BBS), postural control (PASS), activities of daily living (Barthel Index), and patient-reported outcomes (e.g., EQ-5D and satisfaction). We will report effect sizes, minimal clinically important differences, adherence, and safety events, and explore predictors of response (e.g., baseline severity, time since stroke). Phase 2 will extend follow-up to 3, 6, and 12 months to assess the durability of improvements, maintenance strategies (e.g., booster sessions), and real-world participation. Where feasible, we will incorporate economic evaluation (resource use and cost-effectiveness) to inform implementation in routine care.

## 5. Conclusions

In this single-group pretest–posttest study at a single center, a rehabilitation program including Lokomat^®^ training was associated with improvements in balance, postural control, and functional independence. These results support Lokomat^®^ as a component of multidisciplinary neurorehabilitation. Given that we used performance-based outcomes without standardized global disability or patient-reported measures, findings are best interpreted as associations within an integrated program. Future studies will incorporate mRS/NIHSS and EQ-5D, and use controlled designs to extend these observations. These findings support a deliberately integrated rehabilitation model in which Lokomat^®^ training and conventional therapy are aligned to reinforce shared functional goals.

## Figures and Tables

**Table 1:** General sociodemographic and clinical characteristics of the sample.

		Total (*n* = 136)
Age		61.57 ± 1.11
Sex	Male	65 (47.80)
	Female	71 (52.20)
Laterality of the brain injury	Right upper limb	70 (51.50)
	Left upper limb	66 (48.50)
Time after the stroke (months)		35.75 ± 2.27
Diagnosis of stroke	CT	43 (31.6)
	MRI	82 (46.9)
	Both	11 (8.1)
Gait disturbance	Ataxia	5 (3.7)
	Reaper	39 (28.7)
	Festinating Gait	32 (23.5)
	Undefined	36 (26.5)
	No change	10 (7.4)
	No gait	14 (10.3)
Physical activity prior to stroke	Yes	46 (33.8)
	No	90 (66.2)
Medical treatment after stroke	Cranioplasty	8 (5.9)
	Fibrinolysis	68 (50.0)
	Thrombectomy	35 (25.7)
	Craniotomy	15 (11.0)
	Ventricular Drainage	7 (5.1)
	Coil Placement	2 (1.5)
	Anticoagulation	1 (0.7)
Spasticity	Yes	132 (97.1)
	No	4 (2.9)
Comorbidities	Hypertension	27 (19.9)
	Hyperlipidemia	10 (7.4)
	Diabetes Mellitus	19 (14.0)
	Heart Disease	20 (14.7)
	Obesity	22 (16.2)
	Hypothyroidism	17 (12.5)
	Asthma	10 (7.4)
	Neoplasia	6 (4.4)
	Venous Thrombosis	3 (2.2.)
	Epilepsy	2 (1.5)

Values are mean ± SD or *n* (%). SD = standard deviation.

**Table 2:** Preliminary results of the Lokomat.

Outcome	Mean t1	Mean t2	Mean Difference ± SD	% Change	t (df)	*p*-Value	Effect Size Cohen’s d	95% CI of Effect Size
Balance	11.76 ± 13.99	21.91 ± 18.09	−10.15 ± 10.79	+86.3%	−10.980 (135)	0.000	0.94	−11.983 −8.325
Postural Control	15.53 ± 9.23	21.90 ± 9.37	−6.37 ± 4.88	+41.0%	−15.206 (135)	0.000	1.30	−7.196 −5.539
Functional independence	24.71 ± 22.82	41.76 ± 28.04	−17.06 ± 16.39	+69.0%	−12.14 (135)	0.000	1.04	−19.839 −14.279

BBS = Berg Balance Scale (0–56); PASS = Postural Assessment Scale for Stroke Patients (0–36); Barthel = Barthel Index (0–100). Cohen’s d calculated from paired t (dz = t/√*n*). % change rounded to 1 decimal.

## Data Availability

The data shown in this study are available upon request from the corresponding author. The data are not available to the public, since, taking into account the sensitive nature of all the questions asked in this study, all participants were guaranteed that the data obtained would be confidential and would not be shared.

## Abbreviations

The following abbreviations are used in this manuscript:

CVA	Cerebrovascular accident
WHO	World Health Organization
ADL	Activities of daily living
BOS	Base of support
CM	The center of mass
BBS	The Berg Balance Scale
TUG	Timed Up and Go Test
FIM	Functional Independence Measure
PASS	The Postural Assessment Scale for Stroke Patients.
BADL	Basic activities of daily living.
BWS	Body weight support
ROM	Range of motion

## References

[1] Lui F., Khan-Suheb M.Z., Patti L. (2025). Ischemic Stroke. StatPearls.

[2] Katan M., Luft A. (2018). Global Burden of Stroke. Semin. Neurol..

[3] Khan F., Chevidikunnan M.F. (2021). Prevalence of Balance Impairment and Factors Associated with Balance among Patients with Stroke. A Cross Sectional Retrospective Case Control Study. Healthcare.

[4] Li X., He Y., Wang D., Rezaei M.J. (2024). Stroke rehabilitation: From diagnosis to therapy. Front. Neurol..

[5] Rinalduzzi S., Trompetto C., Marinelli L., Alibardi A., Missori P., Fattapposta F., Pierelli F., Currà A. (2015). Balance dysfunction in Parkinson’s disease. Biomed Res. Int..

[6] Lott M.B. (2019). Translating the Base of Support a Mechanism for Balance Maintenance During Rotations in Dance. J. Dance Med. Sci..

[7] Wang J., Li Y., Yang G.Y., Jin K. (2024). Age-Related Dysfunction in Balance: A Comprehensive Review of Causes, Consequences, and Interventions. Aging Dis..

[8] Meskers C.G., Schouten A.C., de Groot J.H., de Vlugt E., van Hilten B.J., van der Helm F.C., Arendzen H.J. (2009). Muscle weakness and lack of reflex gain adaptation predominate during post-stroke posture control of the wrist. J. Neuroeng. Rehabil..

[9] Schoene D., Heller C., Aung Y.N., Sieber C.C., Kemmler W., Freiberger E. (2019). A systematic review on the influence of fear of falling on quality of life in older people: Is there a role for falls?. Clin. Interv. Aging.

[10] Zhang T., Zheng J. (2025). Enhancing postural control in stroke patients: Advances in mechanisms and functional recovery analysis of core stability training. Neurol. Sci..

[11] Kanekar N., Aruin A.S. (2014). The effect of aging on anticipatory postural control. Exp. Brain Res..

[12] Ma C., Chen N., Mao Y., Huang D., Song R., Li L. (2017). Alterations of Muscle Activation Pattern in Stroke Survivors during Obstacle Crossing. Front. Neurol..

[13] Copanitsanou P., Hertz K., Santy-Tomlinson J. (2018). Mobility, Remobilisation, Exercise and Prevention of the Complications of Stasis. Fragility Fracture Nursing: Holistic Care and Management of the Orthogeriatric Patient.

[14] Luna N.M.S., Bobbio T.G., de Graaf M., Greve J.M.D., Ernandes R.d.C., Dias A.S., Lino M.H.d.S., Soares-Junior J.M., Baracat E.C., Mochizuki L. (2024). The decline in postural balance has a negative impact on the performance of functional tasks in individuals with Parkinson’s Disease. Clinics.

[15] Kim J.H., Lee S.B. (2025). Evaluation of Activities of Daily Living: Current Insights and Future Horizons. Ann. Geriatr. Med. Res..

[16] Saso A., Moe-Nilssen R., Mati Gunnes M.G., Askim T. (2016). Responsiveness of the Berg Balance Scale in patients early after stroke. Physiother. Theory Pract..

[17] Saraiva J., Rosa G., Fernandes S., Fernandes J.B. (2023). Current Trends in Balance Rehabilitation for Stroke Survivors: A Scoping Review of Experimental Studies. Int. J. Environ. Res. Public Health.

[18] Todhunter-Brown A., Baer G., Campbell P., Choo P.L., Forster A., Morris J., Pomeroy V.M., Langhorne P., Pollock A., Cochrane Stroke Group (2014). Physical rehabilitation approaches for the recovery of function and mobility following stroke. Cochrane Database Syst. Rev..

[19] Levi M.F., Demers M. (2020). Motor learning in neurological rehabilitation. Disabil. Rehabil..

[20] Sanchez C., Lerma-Lara S., Garcia-Carmona R., Urendes E., Laccourreye P., Raya R. (2022). Studying the Research-Practice Gap in Physical Therapies for Cerebral Palsy: Preliminary Outcomes Based on a Survey of Spanish Clinicians. Int. J. Environ. Res. Public Health.

[21] Masbernat-Almenara M., Hervás S.P., Fernández-Lago H., Gonzalez-Hoelling S., Salgueiro C., Cabanas-Valdés R. (2025). Mapping Physiotherapy Approaches for Stroke Survivors in Catalonia: A Cross-Sectional Study. Rev. De Neurol..

[22] Veerbeek J.M., van Wegen E., van Peppen R., van der Wees P.J., Hendriks E., Rietberg M., Kwakkel G., Quinn T.J. (2014). What is the evidence for physical therapy poststroke? A systematic review and meta-analysis. PLoS ONE.

[23] Kip H., Sieverink F., van Gemert-Pijnen L.J.E.W.C., Bouman Y.H.A., Kelders S.M. (2020). Integrating People, Context, and Technology in the Implementation of a Web-Based Intervention in Forensic Mental Health Care: Mixed-Methods Study. J. Med. Internet Res..

[24] Chang W.H., Kim Y.H. (2013). Robot-assisted Therapy in Stroke Rehabilitation. J. Stroke.

[25] Banyai A.D., Brișan C. (2024). Robotics in Physical Rehabilitation: Systematic Review. Healthcare.

[26] Potašová M., Mačej P., Moraučíková E., Baňárová P.S., Kutiš P. (2025). Lokomat vs. Conventional Therapy—Impact on Gait Symmetry in Hemiparetic Patients: Preliminary Clinical Study. Healthcare.

[27] Gupta A., Prakash N.B., Honavar P.R. (2023). Gait Training with Robotic Exoskeleton Assisted Rehabilitation System in Patients with Incomplete Traumatic and Non-Traumatic Spinal Cord Injury: A Pilot Study and Review of Literature. Ann. Indian Acad. Neurol..

[28] Baronchelli F., Zucchella C., Serrao M., Intiso D., Bartolo M. (2021). The Effect of Robotic Assisted Gait Training with Lokomat^®^ on Balance Control After Stroke: Systematic Review and Meta-Analysis. Front. Neurol..

[29] Pisla D., Nadas I., Tucan P., Albert S., Carbone G., Antal T., Banica A., Gherman B. (2021). Development of a Control System and Functional Validation of a Parallel Robot for Lower Limb Rehabilitation. Actuators.

[30] Vaida C., Rus G., Tucan P., Machado J., Pisla A., Zima I., Birlescu I., Pisla D. (2024). Enhancing Robotic-Assisted Lower Limb Rehabilitation Using Augmented Reality and Serious Gaming. Appl. Sci..

[31] Takeuchi N., Izumi S. (2013). Rehabilitation with poststroke motor recovery: A review with a focus on neural plasticity. Stroke Res. Treat..

[32] Marinaro C., Muglia L., Squartecchia S., Cozza A., Corsonello A., Pranno L., Ferrarin M., Lencioni T. (2025). Mapping the Role of Robot-Assisted Gait Training in Post-Stroke Recovery Among Elderly Patients: A Scoping Review. J. Clin. Med..

[33] Wu L., Xu G., Wu Q. (2023). The effect of the Lokomat^®^ robotic-orthosis system on lower extremity rehabilitation in patients with stroke: A systematic review and meta-analysis. Front. Neurol..

[34] Aurich-Schuler T., Grob F., van Hedel H.J., Labruyère R. (2017). Can Lokomat therapy with children and adolescents be improved? An adaptive clinical pilot trial comparing Guidance force, Path control, and FreeD. J. Neuroeng. Rehabil..

[35] Rodgers H., Bosomworth H., Krebs H.I., van Wijck F., Howel D., Wilson N., Finch T., Alvarado N., Ternent L., Fernandez-Garcia C. (2020). Robot-assisted training compared with an enhanced upper limb therapy programme and with usual care for upper limb functional limitation after stroke: The RATULS three-group RCT. Health Technol. Assess..

[36] Swinnen E., Beckwée D., Meeusen R., Baeyens J.-P., Kerckhofs E. (2014). Does robot-assisted gait rehabilitation improve balance in stroke patients? A systematic review. Top. Stroke Rehabil..

[37] Freixes O., Passuni D.A., Buffetti E., Elizalde M., Lastiri F. (2020). Berg Balance Scale: Inter-rater and intra-rater reliability of the Spanish version with incomplete spinal cord injured subjects. Spinal Cord Ser. Cases.

[38] Valdés R.M.C., Girabent-Farrés D., Cánovas-Vergé F.M., Caballero-Gómez A., Germán-Romero C., Bagur-Calafat C. (2015). Spanish translation and validation of the Postural Assessment Scale for Stroke Patients (PASS) to assess balance and postural control in adult post-stroke patients. Rev. Neurol..

[39] Bernaola-Sagardui I. (2018). Validation of the Barthel Index in the Spanish population. Enferm. Clin..

[40] Papalia G.F., Papalia R., Diaz Balzani L.A., Torre G., Zampogna B., Vasta S., Fossati C., Alifano A.M., Denaro V. (2020). The Effects of Physical Exercise on Balance and Prevention of Falls in Older People: A Systematic Review and Meta-Analysis. J. Clin. Med..

[41] Molteni F., Gasperini G., Gaffuri M., Colombo M., Guanziroli E., Mazzoleni S., Giovanzana C., Lorenzon C., Farina N., Cannaviello G. (2017). Wearable robotic exoskeleton for overground gait training in sub-acute and chronic hemiparetic stroke patients: Preliminary results. Eur. J. Phys. Rehabil. Med..

[42] Liao Y., Yang Y.R., Chen S.J., Wu Y.R., Fuh J.L., Wang R.Y. (2022). Virtual reality–based training to improve obstacle-crossing performance and dynamic balance in patients with Parkinson’s disease. Neurorehabil. Neural Repair.

[43] Sánchez-Herrera B., Martín-González J.M., Pérez-Robledo F. (2018). Efectos de un programa de fisioterapia sensoriomotriz sobre el equilibrio en pacientes con daño neurológico. Rev. Esp. Geriatr. Gerontol..

[44] Oliveira C.B., Medeiros I.R.T., Greters M.E., Frota N.A.F., Lucato L.T., Scaff M., Conforto A.B. (2008). Abnormal sensory integration affects balance control in hemiparetic patients within the first year after stroke. Clinics.

[45] Tsang W.W.N., Hui-Chan C.W.Y. (2005). Effects of exercise on joint sense and balance in elderly men: Tai Chi versus golf. Med. Sci. Sports Exerc..

[46] Promsri A., Pitiwattanakulchai P., Saodan S., Thiwan S. (2024). Age-Related Changes in Postural Stability in Response to Varying Surface Instability in Young and Middle-Aged Adults. Sensors.

[47] Tyson S.F., Connell L.A. (2009). The psychometric properties and clinical utility of measures of walking and mobility in neurological conditions: A systematic review. Clin. Rehabil..

[48] Verheyden G., Nieuwboer A., De Wit L., Thijs V., Dobbelaere J., Devos H., Severijns D., Vanbeveren S., de Weerdt W. (2008). Time course of trunk, arm, leg, and functional recovery after ischemic stroke. Neurorehabilit. Neural Repair.

[49] Lee S.H., Kim H., Lee H.J., Kim K., Lim J.Y. (2021). Effects of robotic trunk rehabilitation on trunk control, balance, and mobility in stroke survivors: A randomized controlled trial. J. Neuroeng. Rehabil..

[50] Calabrò R.S., Naro A., Russo M., Bramanti P. (2022). Shaping neuroplasticity by using powered exoskeletons in patients with stroke: A randomized clinical trial. J. Neuroeng. Rehabil..

[51] Edemekong P.F., Bomgaars D.L., Sukumaran S., Levy S.B. (2025). Activities of Daily Living. StatPearls.

[52] Farzi S., Farzi S., Moladoost A., Ehsani M., Shahriari M., Moieni M. (2019). Caring Burden and Quality of Life of Family Caregivers in Patients Undergoing Hemodialysis: A Descriptive-Analytic Study. Int. J. Community Based Nurs. Midwifery.

[53] Khan F., Amatya B., Galea M.P. (2021). Rehabilitation interventions in adults with multiple sclerosis: A systematic review. Arch. Phys. Med. Rehabil..

[54] Molteni F., Gasperini G., Cannaviello G., Guanziroli E. (2018). Exoskeleton and End-Effector Robots for Upper and Lower Limbs Rehabilitation: Narrative Review. PM R..

[55] Lloréns R., Gil-Gómez J.A., Alcañiz M., Colomer C., Noé E. (2020). Improvement in activities of daily living performance after a virtual reality-based intervention in stroke patients: A randomized controlled trial. Disabil. Rehabil..

[56] Sale P., Franceschini M., Waldner A., Hesse S. (2022). Use of a robotic device in upper limb rehabilitation after stroke: A multicenter study. J. Rehabil. Res. Dev..

